# Editorial: Integrin adhesion receptors in health and disease

**DOI:** 10.3389/fcell.2023.1149920

**Published:** 2023-02-14

**Authors:** Vassiliki Kostourou, Benjamin T. Goult, Andreja Ambriović-Ristov

**Affiliations:** ^1^ Endothelial Pathobiology and tissue, Microenvironment, Athens, Greece; ^2^ School of Biosciences, University of Kent, Canterbury, Kent, United Kingdom; ^3^ Division of Molecular Biology, Ruder Boskovic Institute, Zagreb, Croatia

**Keywords:** adhesion, integrin, extracellular matrix, integrin adhesion complex, integrin signalling

Cells sense the chemical and mechanical properties of their environment. In animals, one mechanism for this sensing is *via* the direct attachment of cells to the surrounding extracellular matrix (ECM). Mediated by the integrin protein family of ECM receptors, integrin adhesion complexes (IACs) engage various ligands of the ECM as well as counter receptors on neighbouring cells. An emerging property of IACs that is essential for tissue homeostasis is their role in cell mechanotransduction, a process whereby cells are able to sense and respond to mechanical cues and convert them into biological signals to elicit different cellular responses. By coupling extracellular ligands to the actin cytoskeleton, microtubules and intermediate filaments, these IACs constitute sites of signalling integration that regulate central cellular functions. The dense, highly dynamic protein assemblies associated with IACs are collectively referred to as the integrin “adhesome” and its mis-regulation is central to a wide variety of developmental and pathological processes. In this Research Topic the full breadth of integrin adhesion biology was explored in a series of 15 articles.

The breadth of research of components of the integrin adhesion complexes IACs was highlighted by the eight Review articles that were included in this Research Topic. All of these were useful and presented comprehensive insight and description of different facets of integrin signalling.

The development of new combined therapies to control the process of wound healing are desperately needed. Perez et al. contributed with a Review paper on the trilogy of Thy-1 (CD90) glycoprotein/integrin/syndecan 4 proteins whose expression is controlled during the healing process. Since lack of expression of any of these proteins results in delayed wound healing, the authors reviewed its interaction as a trimolecular complex in bi-directional signalling that regulates diverse aspects along the stages of the wound healing process.

Thy-1 was also the Research Topic of the Review article by Hu et al. which focused on the ability of Thy-1 to mediate integrin-related signalling through both direct *trans-* and *cis-*interactions with integrins. The article discussed the recent progress and discoveries of Thy-1–integrin interactions in *trans* and in *cis*, highlighting their pathophysiological consequences and exploring other potential binding partners of Thy-1 within the integrin regulation/signalling paradigm.

Bergonzini et al. wrote an informative review on disappointments and opportunities of targeting integrins for cancer therapy. Dysregulation of integrin functions has been extensively reported in cancer and associated with tumour growth, invasion, angiogenesis, metastasis, and therapy resistance. However, despite encouraging preclinical data, targeting integrin adhesion complexes in clinical trials has thus far failed. The authors systematised excellent the contributing factors to therapeutic failure and provided an overview of emerging, promising approaches that are being investigated to use integrins as prognostic biomarkers and to improve therapeutic delivery at the tumour site *via* integrin binding.

The co-operation of integrins with other signalling pathways in cancer was highlighted in a comprehensive review by Stanislovas and Kermogrant which discussed the interplay between c-MET receptors and integrins. Accumulating evidence derived from cellular assays, *in vivo* and in human tissue studies, shows different modes of co-operation between integrin receptors and c-MET activation, with clinical implications. Integrin binding to ECM can activate c-MET signalling and c-MET activation can increase integrin-mediated cell adhesion and migration. Notably, independently of cell adhesion, cooperation of c-MET with integrins in endocytic vesicles promote anchorage-independent survival. Collectively, the c-MET-integrin signalling axis promotes cancer and metastasis and thus provides novel therapeutic interventions.

The importance of cell-substratum adhesion in regulating cellular function is not only limited to mammalian cells. Both Amoebozoa and Metazoa have cell adhesion structures but they lack integrins. Mijanovic and Weber offer a detailed overview of the cell anchorage machinery in *Dictyostelium* amoebae highlighting the similarities with integrin-mediated adhesions in animal cells. The short-lived and less-specific interactions of *Dictyostelium* adhesion sites are pertinent to the mesenchymal to amoeboid transition that drives metastasis.

The Review article by Sun et al. was timely in that it summarised a very active area of research. Rap1 is the main GTPase that activates the protein talin-1. Yet, there are a number of direct and indirect mechanisms by which Rap1 achieves this. In some systems Rap1 binding directly to talin-1 is required, but in other systems, the Rap1 effector RIAM, bridges Rap1 to talin-1. The figure they present, summarising how integrin activation pathways differ by cell type, will be useful to many researchers because it clearly highlights how the RIAM (and Lamellipodin)-dependent pathways and the direct binding of Rap1 to talin-1 coexist and contribute in parallel in blood cells, including immune cells.

Another important player in the regulation of integrin complexes is the protein paxillin. Despite its ubiquity in integrin adhesions and it being an essential gene, the precise role paxillin plays has not been fully deciphered. The Review article by Ripamonti et al. offered an overview of the molecular bases of the mechano-sensitivity and mechano-signalling capacity of paxillin as a key component of the mechano-transducing machinery.

Building on the mechanobiology of integrin signalling, the Review article by Banerjee et al. provides a useful insight into the role of mechanical forces in diverse immune cell processes and their dysregulation during autoimmune disorders. This comprehensive review and its informative figures will provide a useful reference for researchers interested in the role of integrins in immunity.

As well as a series of timely reviews, the Research Topic includes seven Original research articles that show the wide utility of integrins in health and disease.

The Original research article by Keramidioti et al. focuses on epithelial morphogenesis in the *Drosophila* egg chamber and the role of integrin adhesome components, Parvin and ILK, in epithelia dynamic reorganisation. They demonstrated that Parvin and ILK are required in pre-follicle cells for germline cyst encapsulation and stalk cell morphogenesis. In contrast, although the preservation of the monolayer organisation in the middle stage egg chambers termini requires integrins, it does not require Parvin or ILK. Collectively, their data uncovered novel developmental functions for both Parvin and ILK, which closely synergize with integrins in epithelia.

The Original research article by Valencia-Expósito et al. used the *Drosophila* wing imaginal disc epithelium to evaluate the importance of integrins as survival factors during epithelia morphogenesis. Attachment of cells to the ECM is required for cell survival, and disruption of this interaction leads to a specific type of apoptosis known as anoikis. However, during development some cells need to detach, so mechanisms need to be in place to suppress anoikis in these cells. Their results indicate that, during wing disc morphogenesis, the EGFR signalling pathway provides survival signals that protect cells undergoing cell shape changes which require detachment from the ECM from triggering anoikis. They suggest that the cooperation of integrin signalling with the EGFR/Ras signalling pathways enables cell survival during morphogenesis.

The role of integrin signalling in epithelial cells during collective cell migration was thoroughly examined in the Original research article by Hight-Warburton et al. The authors investigated the role of ɑ4β1 and ɑ9β1 integrins, known to regulate wound healing responses. Using pharmacological inhibitors, they showed that co-localisation of ɑ4β1 and ɑ9β1 integrins in the migratory front of the keratinocyte monolayer controls cell cytoskeletal dynamics, migration and proliferation through local suppression of Mitogen and Stress Activated Kinase 1 (MSK1) and ERK1/2 activation. These studies provided novel mechanistic insight into integrin signalling during collective cell migration with therapeutic relevance.

Integrin signalling is also regulated by the surface availability of integrin receptors. Several studies have shown that integrin endocytosis and recycling regulate cellular function. In an elegant Original research article, Meecham et al., investigated the fate of ligand-bound ɑvβ6 internalisation and its effect on cell migration. Using a novel flow cytometry-based RNAi screen, they demonstrated that both clathrin and caveolin mediate ligand-bound ɑvβ6 endocytosis and revealed key molecular players in integrin trafficking. Importantly for the clinic, they showed that the internalised ligand-bound ɑvβ6 integrin is not degraded, but to a great extent it gets recycled to the cell surface, thus facilitating the design of ɑvβ6-based therapeutics for cancer and fibrosis.

The crosstalk between different cell adhesive structures is crucial to correct cellular functioning. The Original research article by Schmidt et al. looked at the interplay of different integrin-mediated adhesion structures, namely, the α6β4-integrin mediated hemidesomosomes and the β1-integrin mediated Focal adhesions. The loss of hemidesmosomes has been reported in various cancers such as prostate cancer and has been shown to correlate with increased invasive migration. The authors showed that knocking down α6β4-integrins promoted collective cell migration and modulated migratory activity of prostate epithelial cells. The authors analysis indicated that focal adhesion kinase (FAK) was involved in this changed cellular behaviour.

The importance of hemidesmosomes in regulating cell behaviour was further investigated in the Original research article by Tadijan et al. who looked at the factors that led to cancer drug resistance in a tongue squamous cell carcinoma model. Using Mass spectrometry -based proteomics analysis, immunofluorescence and electron microscopy, the authors identify a key role for α6β4-containing type II hemidesmosomes in regulating anticancer drug sensitivity. The causative relationship between integrin expression and resistance to anticancer drugs has been demonstrated in different tumors, including head and neck squamous cell carcinoma and hemidesmosomes seem to be playing an important role.

Integrin interactions with different adhesive structures and morphogenic factors can also facilitate cell differentiation. In the Original research article from Valat et al., BMP-2 was shown to trigger myoblast differentiation to osteoblasts by modulating the interaction of specific integrin subunits with cadherins and activating specific transcription pathways.

In summary, the Research Topic identifies the vibrancy of the Integrin field, and the ever-expanding role of integrins in coordinating cellular processes. The next decade promises to be very exciting.

